# Psychometric properties of patient‐reported outcomes measures used to assess upper limb pathology: a systematic review

**DOI:** 10.1111/ans.17973

**Published:** 2022-08-12

**Authors:** Samuel Abbot, Susanna Proudman, Yih Ping Sim, Nicole Williams

**Affiliations:** ^1^ Department of Orthopaedics and Trauma The Queen Elizabeth Hospital Adelaide South Australia Australia; ^2^ Department of Orthopaedics and Trauma Royal Adelaide Hospital Adelaide South Australia Australia; ^3^ Department of Orthopaedics and Trauma Women's and Children's Hospital Adelaide South Australia Australia; ^4^ Discipline of Medicine University of Adelaide Adelaide South Australia Australia; ^5^ Department of Orthopaedics and Trauma Lyell McEwin Hospital Adelaide South Australia Australia; ^6^ Centre for Orthopaedic and Trauma Research University of Adelaide South Australia Australia

**Keywords:** construct validity, content validity, orthopaedic surgery, psychometric properties, upper limb

## Abstract

**Background:**

With the continued development of patient‐centred healthcare models, patient‐reported outcome measures (PROMs) are increasingly used to evaluate outcomes in patients with upper limb pathology. The aim was to identify valid, reliable and responsive PROMs used to assess outcomes following upper limb pathology, and ascertain how their psychometric properties had been established. A secondary aim was to identify PROMs that have been validated to assess upper limb pathology in the paediatric population.

**Methods:**

A review of the Medline and EMBASE database was performed. Articles that analysed the validity of an established PROM used for upper limb pathology were included. Extracted study data included: author, country, PROM(s) investigated, year of publication, study type, sample size, demographics and duration of follow‐up.

**Results:**

Twenty‐five articles were included, which together investigated the psychometric properties of 23 different PROMs that have been used to assess outcomes in adults following upper limb pathology. No study evaluated the psychometric properties of PROMs used in the paediatric population. Among PROMs that have been used in adults, the Quick‐Disabilities of the Arm, Shoulder and Hand (QuickDASH) had strong content‐ and construct‐validity, reliability and responsiveness in comparison to others.

**Conclusion:**

There are currently no studies that have analysed the content validity of PROMs used to assess upper limb pathology in the paediatric population. Prospective studies are required for the development of PROMs that can be utilized in children to assess upper limb pathology.

## Graphical abstract

A systematic literature review analysing the psychometric properties of patient‐reported outcome measures that have been used to assess upper limb pathology in children. Prospective studies in children and adolescents are required to determine which patient‐reported outcome measures are valid and reliable to be used in the paediatric population.

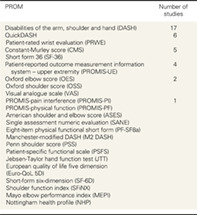

## Introduction

With the continued development of patient‐centred healthcare models, patient‐reported outcome measures (PROMs) are increasingly used to evaluate outcomes in patients with upper limb pathology.^1^ PROMs allow for an insight into a patient's subjective experience of their health condition and its therapy, and are required to evaluate functional and quality‐of‐life outcomes.^2^ A large variety of PROMs have been utilized in orthopaedic research, and it can be challenging to ascertain which PROM is most suitable for a given population and condition. The ideal PROM must be valid, reliable and responsive to change in clinical status, without being overly arduous to complete.^1^, ^3^ While numerous studies have investigated the psychometric properties of PROMs used to assess upper limb pathology in adults, relatively few studies have evaluated their use in the paediatric population. As previous studies have demonstrated, it is important to evaluate the psychometric properties of PROMs prior to implementing their clinical use in the target population.^1^ The aim of this systematic review was to identify valid, reliable and responsive PROMs that have been used to assess upper limb pathology, and ascertain how their psychometric properties had been established. A secondary aim was to identify PROMs that have been validated to assess upper limb pathology in children and adolescents.

## Materials and methods

This systematic literature review was conducted in accordance with the preferred reporting items for systematic reviews and meta‐analyses (PRISMA) guidelines.^4^

### Definitions

Content validity refers to the degree to which elements of a PROM are relevant to a representative of the targeted construct for a particular assessment purpose.^5^ It addresses whether a questionnaire has enough items to cover the area of interest adequately, and whether it measures important elements of the pathology for which it is used.^6^ This is distinct from construct validity, which is established by examining the relationships between the PROM of interest and other instruments that are expected to be related, and can be measured using convergent and divergent validity approaches.^7^ Reliability refers to the reproducibility of scores from one assessment to another, and is usually expressed in the forms of internal consistency (measured as Cronbach's alpha) and test–retest reliability (measured as the intraclass coefficient, ICC).^8^ Responsiveness refers to the ability of a PROM to distinguish important clinical change from measurement error, and is a measure of longitudinal validity.^3^ The most commonly reported measures of responsiveness are effect size (ES) and standardized response mean (SRM). Floor and ceiling effects are another important consideration when analysing the psychometric properties of a PROM. They are considered to be present if more than 15% of participants achieve the lowest or highest possible score, indicating that further impairment or improvement cannot be detected in these patients, respectively.^9^ If present, floor and ceiling effects are detrimental to the content validity, reliability and responsiveness of a PROM.^3^

### Eligibility criteria

Included articles were original research studies written in English that analysed the validity of an established PROM used for pathology of the upper limb. There was no restriction on publication date. Articles were excluded if they did not mention how the validity of the PROM was established.

### Search strategy

A librarian‐assisted search was performed on the Medline and Embase databases on fourth December 2021 (see Files S1 and S2). The search syntax consisted of four categories of keywords and/or subject headings. These categories were: terms related to (i) upper limb pathology; (ii) outcome measures; (iii) psychometric properties and (iv) paediatrics. Articles with a relevant title and abstract were read in full text by two authors (SA and YPS), and those that fulfilled the eligibility criteria were selected. Discrepancies in selection between the two authors were resolved by discussion.

### Data collection

The included articles were reviewed for established PROMs that have been used for upper limb pathology, the psychometric properties of the PROMs that were analysed (including validity, responsiveness and reliability), and the method by which the psychometric properties of the PROM were established. The study data extracted were author, country, PROM(s) investigated, year of publication, study type, sample size, demographics and duration of follow‐up.

## Results

### Study selection

**Fig. 1:**
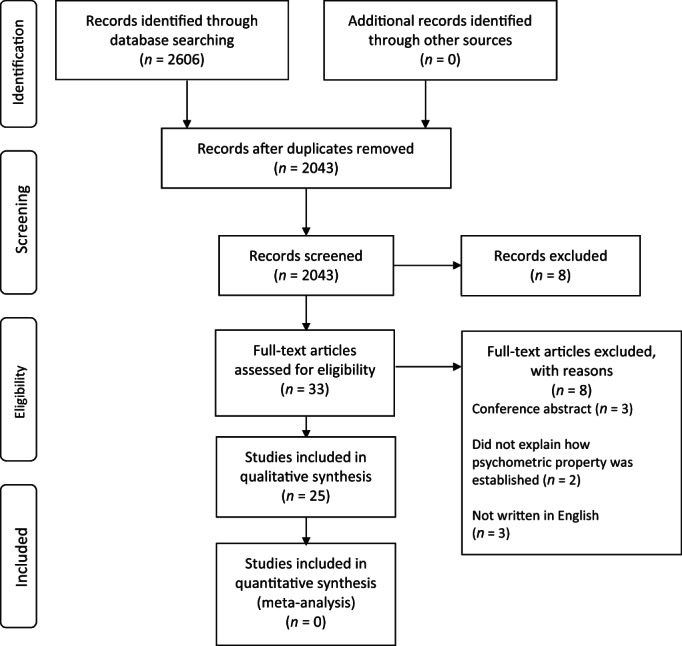
PRISMA flow diagram depicting the process by which articles were screened.

The search yielded 2606 results, of which there were 563 duplicates. Therefore, 2043 titles were screened. Thirty‐three articles were read in full text after studies were excluded based on title and abstract. Eight were excluded on full‐text review for the following reasons: did not sufficiently explain how the discussed psychometric properties were established (two), conference presentations (three) and non‐English language (three). Thus, 25 articles were finally selected for inclusion. This is outlined in the PRISMA diagram (Fig. 1). There was not a single article yielded by the search strategy that evaluated the validity of PROMs used for assessing upper limb pathology in the paediatric population; all 25 eligible articles evaluated the psychometric properties of PROMs used in adults.

### Study design

All 25 articles were prospective cohort studies. There was a total of 4575 participants with a mean age of 54.4 years (*SD* = 8.8 years); 61% were female and the median follow‐up was 9 months (range 5 days–3.3 years). Fifteen studies examined content validity, 23 calculated construct validity, 19 assessed reliability and 12 examined the responsiveness of the PROMs that they reviewed.

### PROMs discussed

Twenty‐three reported PROMs were identified (Table 1). The 30‐item, patient‐reported disabilities of the arm, shoulder and hand (DASH) questionnaire was the most commonly investigated PROM, being reviewed in 17 studies.^10^ The DASH is designed to measure a patient's perception of their physical function, symptoms, quality of life and ability to conduct activities of daily living (ADLs) in relation to their upper extremity pathology.^1^ Development of this PROM allowed for the disability experienced by patients affected by upper limb pathology to be estimated, and for any changes in their symptoms and perceived functional status to be monitored over time.^11^ It has been shown that the DASH is an effective tool in these roles and can be used for research purposes and clinical practice.^11^, ^12^ The shortened, 11‐item version of the DASH, namely the ‘QuickDASH’, was reviewed in six studies.^13^ The items in the QuickDASH were selected from the original tool on the basis of them having the highest reliability, validity and responsiveness within each domain of the DASH.^12^ There are seven questions related to the patient's ability to perform ADLs that involve the upper limb, three questions related to pain and paraesthesia and one question related to social functioning. The Constant‐Murley Score (CMS) was discussed in five studies.^14^ Since its inception in 1987, the CMS has been widely utilized to evaluate overall shoulder function in research related to upper limb pathology. The CMS is a combined patient‐ and clinician‐reported outcome measure, consisting of four domains: pain, ADLs, range of motion (ROM) and strength.^1^ The patient is given a score out of 100, with a higher score being indicative of better shoulder function. The patient‐rated wrist evaluation (PRWE) is a 15‐item PROM specific to wrist‐related pain and disability, and was reviewed in six studies.

**Table 1:** Patient‐reported outcome measures (PROMs) discussed within the included articles

PROM	Number of studies
Disabilities of the arm, shoulder and hand (DASH)	17
QuickDASH Patient‐rated wrist evaluation (PRWE)	6
Constant‐Murley score (CMS) Short form 36 (SF‐36)	5
Patient‐reported outcome measurement information system – upper extremity (PROMIS‐UE)	4
Oxford elbow score (OES) Oxford shoulder score (OSS) Visual analogue scale (VAS)	2
PROMIS‐pain interference (PROMIS‐PI) PROMIS‐physical function (PROMIS‐PF) American shoulder and elbow score (ASES) Single assessment numeric evaluation (SANE) Eight‐item physical functional short form (PF‐SF8a) Manchester‐modified DASH (M2 DASH) Penn shoulder score (PSS) Patient‐specific functional scale (PSFS) Jebsen‐Taylor hand function test (JTT) European quality of life five dimension (Euro‐QoL 5D) Short‐form six‐dimension (SF‐6D) Shoulder function index (SFiNX) Mayo elbow performance index (MEPI) Nottingham health profile (NHP)	1

### Psychometric properties of PROMs

#### Disabilities of the arm, shoulder and hand

Fourteen of the seventeen studies that reviewed the DASH concluded that it has strong psychometric properties and is appropriate to use in clinical research and practice.^6^, ^7^, ^9^, ^15^, ^16^, ^17^, ^18^, ^19^, ^20^, ^21^, ^22^, ^23^, ^24^, ^25^ The convergent validity of the DASH with other PROMs was, in all cases, statistically significant and moderate to very strong. The DASH was calculated to have a Pearson's correlation coefficient of 0.57 with the Short Form 36 (*P* < 0.001),^9^ 0.60 with the Nottingham Health Profile,^6^–0.78 with the Shoulder Function Index (SFinx),^22^ 0.85 with the Oxford Shoulder Score,^23^ and 0.96 with the QuickDASH.^15^ Three studies found a strong correlation between the DASH and the PRWE, with Spearman's correlation coefficient values ranging between 0.59 and 0.90 (*P* < 0.01).^7^, ^19^, ^21^ The DASH also had a strong Spearman's correlation coefficient with the Patient‐Reported Outcome Measurement Information System – Upper Extremity (PROMIS‐UE), Manchester‐Modified DASH (M2‐DASH) and patients' overall self‐assessment of shoulder function, with Spearman's correlation coefficient values of 0.79, 0.98 and −0.7, respectively.^9^, ^20^, ^26^ Four studies criticized the DASH for not having satisfactory content validity when analysing the instrument individually. Two studies used Rasch analysis to determine that the DASH is not a unidimensional scale.^27^, ^28^ In keeping with this, Van de Water *et al*. compared items within the DASH to the International Classification of Functioning, Disability and Health (ICF) and found that multiple items refer to several different domains of health, rather than the single construct of shoulder ‘function’.^23^ Similarly, Khan *et al*. concluded that the DASH is not specific to upper limb pathology, after finding that a group of patients with lower limb pathology had significantly higher DASH scores than a healthy control group.^26^ In contrast, Van Eck *et al*. and Van Leishout *et al*., in their relatively large studies of 370 and 400 participants respectively, concluded that the items of the DASH assess a unidimensional trait, being ‘disability’, based on confirmatory factor analysis and Rasch analysis.^24^, ^25^ No significant floor or ceiling effect for the DASH was identified in any study. The internal consistency of the DASH was excellent in all studies that analysed its reliability, with Cronbach's alpha values ranging between 0.96 and 0.97, indicating high correlation among the 30 items.^6^, ^16^, ^17^, ^26^ Good to excellent test–retest reliability was also found for the DASH, with ICC ranging between 0.83 and 0.97, indicating a strong degree of concordance. The responsiveness of the DASH was acceptable, with ES values between 0.5 and 1.39, and SRM between 1.51 and 2.13.^6^, ^9^, ^17^, ^21^, ^23^ The ES and SRM values were proportional to the duration of follow‐up of the various studies that analysed the longitudinal validity of this instrument.

#### QuickDASH

All six studies that analysed the QuickDASH concluded that it has strong psychometric properties.^2^, ^3^, ^15^, ^21^, ^29^, ^30^ It had strong convergent validity with the single assessment numeric evaluation (SANE) and PROMIS‐UE, with Spearman's correlation coefficients, *r*, of −0.73 and − 0.8, respectively (*P* < 0.05).^3^ A high correlation was found with the PRWE (*r* = 0.81), the PROMIS‐Physical Function (PROMIS‐PF) (*r* = −0.83; *P* < 0.001), the eight‐Item Physical Functional Short Form (PF‐SF8a) (*r* = −0.79) and patients' perceived handicap (*r* = 0.79) and activities of daily living (ADL) scores (*r* = −0.73).^15^, ^21^, ^29^, ^30^ The QuickDASH had excellent reliability, with Cronbach's alpha coefficient ranging between 0.89 and 0.93, and an ICC of 0.94.^15^, ^30^ All three studies that analysed the sensitivity of the QuickDASH to clinical improvement found that it has excellent responsiveness, with SRM values ranging between 1.09 and 2.17, and an ES of 1.23.^3^, ^15^, ^21^

#### Constant‐Murley score

The five studies that reviewed the psychometric properties of the CMS found that it has moderate to high convergent validity with other legacy PROMs. The CMS had Spearman's correlation coefficients with the DASH ranging between −0.78 and − 0.86, with overall pain scores of −0.52, and with ADL scores of 0.72.^17^, ^23^ Similarly, the CMS had a high level of concordance with novel PROMs, with a Pearson's correlation coefficient of 0.87 with the Penn Shoulder Score, and 0.89 with the SFInX (*P* < 0.01).^22^, ^31^ Mahabier *et al*. found that the internal consistency of the CMS was significantly less than the DASH, with a Cronbach's alpha of 0.61.^17^

#### Patient‐rated wrist evaluation

The PRWE was considered to have strong psychometric properties in five of the six studies that reviewed it. One study criticized this instrument for having a significant ceiling effect, with 12% of participants having the highest possible score at final follow‐up.^29^ As discussed earlier, the PRWE correlated strongly with DASH and QuickDASH scores, and was also found to have strong convergent validity with the SF‐36 subscale for bodily pain (*r* = 0.58).^7^ Low correlations were found with other components of the SF‐36; for example, with the physical‐functioning subscale (*r* = 0.31).^7^ There was unanimous agreement that the PRWE is a reliable instrument, with ICC values ranging between 0.90 and 0.94, and Cronbach alpha values ranging between 0.85 and 0.89.^7^, ^16^, ^18^, ^19^ Tsang *et al*. also found that the PRWE is highly responsive to clinical change, with a SRM of 2.19.^21^

## Discussion

In order to be used to assess a clinical condition, a PROM must have content validity, meaning it needs to address important elements of the pathology and its impact on a patient's quality of life.^3^ As demonstrated by the findings of this systematic review, there is a plethora of instruments that have been developed to assess treatment and quality‐of‐life outcomes following upper limb pathology. However, many of these were designed for use in patients with rotator cuff pathology or symptoms of instability, so selecting a PROM for use in a paediatric population following acute trauma is problematic.^1^ The fact that there was no study yielded by our search strategy that assessed the psychometric properties of PROMs used in the paediatric population demonstrates the lack of evidence supporting their use in children and adolescents.

The DASH was, for the most part, found to have strong psychometric properties when utilized in adults. While the DASH had moderate to excellent convergent validity with a large variety of PROMs in numerous studies, there is a possibility of a significant publication bias, as many of these studies were designed to assess the quality of novel PROMs, using the DASH as a comparator.^7^, ^16^, ^18^, ^19^, ^22^ Interestingly, multiple studies that investigated the content validity of the DASH in isolation concluded that it does not satisfy the assumptions of Rasch analysis, in that it was not found to be a unidimensional scale.^27^, ^28^ As the DASH is intended to assess a single trait, being ‘disability’, the items of the DASH should be unidimensional. Furthermore, a group of patients suffering from lower limb pathology in a well‐designed case–control study were found to have significantly higher DASH scores than a healthy control group, suggesting that the DASH is not specific to upper limb pathology.^26^ As has been demonstrated in previous studies, PROMs that are more specific to the pathology that they are intended to assess tend to be more responsive to clinical change. The PRWE, a PROM that is highly specific to symptoms related to the wrist and hand, was found to be more responsive than the DASH in assessing clinical improvement over time in patients following fracture of the distal radius.^21^ The logical explanation is that those questionnaires which target a certain body region are more able to detect symptoms related to that body part's pathology.^6^ Despite its wide acceptance and frequent use, certain concerns regarding the psychometric properties of the CMS have been noted in numerous studies, with its item selection criteria, reliability and validity being suboptimal.^16^ In their prospective cohort study of 140 participants with humeral shaft fractures, Mahabier *et al*. found reliability for the DASH, with a Cronbach's alpha value of 0.96, but not for the CMS (Cronbach's alpha = 0.61).^17^

On the other hand, the QuickDASH was found to have strong content‐ and construct‐validity in all studies that reviewed its psychometric properties when used in adults, and had excellent correlation with the full‐length DASH, as well as patients' perceived handicap and ADL scores.^15^, ^21^ It was also found in multiple studies to have excellent reliability and responsiveness in comparison to other PROMs.^3^, ^15^, ^21^ The strong correlation of the 11‐item QuickDASH with the full‐length instrument suggests that it could be the preferred scale, as it is significantly shorter and the questions are more specific to upper limb pathology.^15^ Additionally, the questions are more appropriate for use in children, as items in the full‐length DASH related to adult functions such as sexual activities and the ability to change a lightbulb, have been excluded in the QuickDASH. Notwithstanding this, multiple items within the QuickDASH assess activities that are not routinely carried out by children, such as ‘doing heavy household tasks’ and ‘using a knife’. Similarly, it enquires about symptoms that would likely challenge the recall of children under the age of 11 years, such as ‘tingling’ in the affected arm. The language used in this questionnaire would also need to be simplified prior to its implementation for use in children. For example, the instruction to ‘rate your ability to do the following activities in the last week’ could be abbreviated to ‘please say whether these tasks are hard for you’.

Examples of legacy PROMs that have been well‐validated in children include the Paediatric Outcomes Data Collection Instrument (PODCI) and the Child Health Questionnaire (CHQ).^32^, ^33^ While these are general health‐related quality‐of‐life questionnaires, rather than being specific to a certain pathology, they exemplify the language and choice of items that are required for a successful paediatric instrument. The PODCI exists in three versions: a questionnaire for children under the age of 11 years (completed by the caregiver) and two surveys for adolescents aged 11 years and older (one completed by the adolescent and one by their caregiver). The CHQ exists in both parent‐reported forms (CHQ‐PF50 and CHQ‐PF28), which are normed for children aged 5–18 years, as well as child‐reported forms (CHQ‐CF87 and CHQ‐CF45), which are normed for children aged 8–18 years. The items included in the PODCI and CHQ to assess the child's health‐related quality of life are quite similar and are relatable for children from a young age. They enquire about the child's ability to run, dress themselves and climb stairs, for example. The language utilized in the CHQ‐CF45 has been validated for use in children as young as the second grade.^34^ It asks in lay terms about the child's ability to ‘get schoolwork done’ and to ‘get along with others’, while the PODCI asks how often the child has been able to ‘get together and do things with friends’. Constructing a questionnaire with this simplified language is one way of negotiating the variable linguistic capabilities of younger children and allowing them to provide a meaningful response.

There is a pressing need for concise yet robust self‐reported PROMs that can be used in children to capture their health‐related physical, emotional and social wellbeing.^34^ There are multiple factors that challenge the development of such PROMs, including age‐related vocabulary, comprehension of health concepts and determining the lower age limit at which children can provide valid and reliable responses.^35^ Despite these challenges, the lack of a valid outcome measure that can be utilized to assess outcomes following upper limb pathology in children represents a significant gap in the literature. Prospective studies are required to assess whether modified versions of adult PROMs, or entirely novel paediatric PROMs, may be effective in assessing upper limb pathology in the paediatric population.

## Conclusion

In conclusion, there are no studies that have analysed the content validity of PROMs used to assess upper limb pathology in the paediatric population. Upon analysis of studies reviewing the use of PROMs in adults, the QuickDASH and PRWE have been demonstrated to have strong psychometric properties. Prospective studies in children and adolescents are required to develop PROMs that may be used to assess upper limb pathology in this population.

## Conflict of interest

None declared.

## Author contributions

**Samuel Abbot:** Conceptualization; data curation; formal analysis; investigation; project administration; writing – original draft; writing – review and editing. **Susanna Proudman:** Supervision; validation; writing – review and editing. **Yih Ping Sim:** Data curation; formal analysis. **Nicole Williams:** Conceptualization; methodology; supervision; writing – review and editing.

## Supporting information


**Supplementary File S1** Search strategy used for Embase.

**Supplementary File S2** Search strategy used for Medline.
