# Supplementary material for: Psychometric properties of patient‐reported outcomes measures used to assess upper limb pathology: a systematic review
Source: ANZ J Surg. 2022 Aug 12;92(12):3170–5. doi: 10.1111/ans.17973 (PMC10087017; doi:10.1111/ans.17973)
Supplement: Supplementary file 1 — Supplementary File S1 Search strategy used for Embase. [file ANS-92-3170-s002.docx]

**Supplementary File 1:** Search strategy used for Embase

Database: Embase <1974 to 2021 December 04>

Search Strategy:

--------------------------------------------------------------------------------

1 Humeral Fractures/ (6461)

2 Shoulder Fractures/ (731)

3 (proximal humer* adj5 fracture*).mp. (3974)

4 (shoulder adj5 fracture*).mp. (3496)

5 (exp Upper Extremity/ or upper limb.mp.) and fracture*.mp. (21577)

6 1 or 2 or 3 or 4 or 5 (30260)

7 functional outcome*.mp. (69478)

8 "Surveys and Questionnaires"/ (750398)

9 "Quality of Life"/ (509628)

10 (quality adj1 life).mp. (17831)

11 (screen* or assess* or test* or surveill* or survey* or questionnaire* or scale* or score* or measur* or instrument* or index* or function*).mp. (18130765)

12 patient reported outcome*.mp. (49235)

13 patient reported outcome measures/ (28220)

14 7 or 8 or 9 or 10 or 11 or 12 or 13 (18247002)

15 Mayo elbow performance.mp. (1334)

16 (Disabilities of the arm, shoulder and hand).mp. [mp=title, abstract, heading word, drug trade name, original title, device manufacturer, drug manufacturer, device trade name, keyword, floating subheading word, candidate term word] (5736)

17 (American Shoulder and Elbow Surgeons Standardized Shoulder Assessment*).mp. [mp=title, abstract, heading word, drug trade name, original title, device manufacturer, drug manufacturer, device trade name, keyword, floating subheading word, candidate term word] (119)

18 (Shoulder pain and disability index*).mp. [mp=title, abstract, heading word, drug trade name, original title, device manufacturer, drug manufacturer, device trade name, keyword, floating subheading word, candidate term word] (894)

19 Simple shoulder test*.mp. (990)

20 Western ontario shoulder instability.mp. (278)

21 (Constant-Murley score* or Constant Murley score or Child Health Questionnaire or PODCI or Pediatric Outcomes Collection Instrument or SPADI or Neer shoulder score or Visual Analogue Scale or VAS).mp. (107871)

22 Shoulder disability questionnaire*.mp. (101)

23 Oxford shoulder score.mp. (484)

24 Elbow self-assessment score*.mp. (6)

25 Morrey elbow score*.mp. (27)

26 15 or 16 or 17 or 18 or 19 or 20 or 21 or 22 or 23 or 24 or 25 (114655)

27 (longitudinal construct validity or valid* or reliab* or responsiveness or content validity).mp. (1841152)

28 exp "reproducibility of results"/ (229066)

29 27 or 28 (1976800)

30 14 or 26 (18254789)

31 6 and 29 and 30 (1730)

32 (paediatric or pediatric or child* or minor or infant or baby or babies or juvenile*).mp. [mp=title, abstract, heading word, drug trade name, original title, device manufacturer, drug manufacturer, device trade name, keyword, floating subheading word, candidate term word] (3590951)

33 31 and 32 (240)

***************************
