# Supplementary material for: Psychometric properties of patient‐reported outcomes measures used to assess upper limb pathology: a systematic review
Source: ANZ J Surg. 2022 Aug 12;92(12):3170–5. doi: 10.1111/ans.17973 (PMC10087017; doi:10.1111/ans.17973)
Supplement: Supplementary file 2 — Supplementary File S2 Search strategy used for Medline. [file ANS-92-3170-s001.docx]

**Supplementary File 2:** Search strategy used for Medline

Database: Ovid MEDLINE(R) ALL <1946 to December 04, 2021>

Search Strategy:

--------------------------------------------------------------------------------

1 Humeral Fractures/ (7763)

2 Shoulder Fractures/ (3487)

3 (proximal humer* adj5 fracture*).mp. (3050)

4 (shoulder adj5 fracture*).mp. (4633)

5 (exp Upper Extremity/ or upper limb.mp.) and fracture*.mp. (8296)

6 1 or 2 or 3 or 4 or 5 (19619)

7 functional outcome*.mp. (47843)

8 "Surveys and Questionnaires"/ (495833)

9 "Quality of Life"/ (212236)

10 (quality adj1 life).mp. (8642)

11 (screen* or assess* or test* or surveill* or survey* or questionnaire* or scale* or score* or measur* or instrument* or index* or function*).mp. (13661259)

12 patient reported outcome*.mp. (26635)

13 patient reported outcome measures/ (8411)

14 7 or 8 or 9 or 10 or 11 or 12 or 13 (13706382)

15 Mayo elbow performance.mp. (1129)

16 (Disabilities of the arm, shoulder and hand).mp. [mp=title, abstract, original title, name of substance word, subject heading word, floating sub-heading word, keyword heading word, organism supplementary concept word, protocol supplementary concept word, rare disease supplementary concept word, unique identifier, synonyms] (3520)

17 (American Shoulder and Elbow Surgeons Standardized Shoulder Assessment*).mp. [mp=title, abstract, original title, name of substance word, subject heading word, floating sub-heading word, keyword heading word, organism supplementary concept word, protocol supplementary concept word, rare disease supplementary concept word, unique identifier, synonyms] (110)

18 (Shoulder pain and disability index*).mp. [mp=title, abstract, original title, name of substance word, subject heading word, floating sub-heading word, keyword heading word, organism supplementary concept word, protocol supplementary concept word, rare disease supplementary concept word, unique identifier, synonyms] (555)

19 Simple shoulder test*.mp. (868)

20 Western ontario shoulder instability.mp. (224)

21 (Constant-Murley score* or Constant Murley score or Child Health Questionnaire or PODCI or Pediatric Outcomes Collection Instrument or SPADI or Neer shoulder score or Visual Analogue Scale or VAS).mp. (68584)

22 Shoulder disability questionnaire*.mp. (81)

23 Oxford shoulder score.mp. (395)

24 Elbow self-assessment score*.mp. (6)

25 Morrey elbow score*.mp. (22)

26 15 or 16 or 17 or 18 or 19 or 20 or 21 or 22 or 23 or 24 or 25 (73369)

27 (longitudinal construct validity or valid* or reliab* or responsiveness or content validity).mp. (1335151)

28 exp "reproducibility of results"/ (420175)

29 27 or 28 (1564201)

30 14 or 26 (13713728)

31 6 and 29 and 30 (906)

32 (paediatric or pediatric or child* or minor or infant or baby or babies or juvenile*).mp. [mp=title, abstract, original title, name of substance word, subject heading word, floating sub-heading word, keyword heading word, organism supplementary concept word, protocol supplementary concept word, rare disease supplementary concept word, unique identifier, synonyms] (3395247)

33 31 and 32 (164)

***************************
